# Plant‐facilitated effects of exotic earthworm *Pontoscolex corethrurus* on the soil carbon and nitrogen dynamics and soil microbial community in a subtropical field ecosystem

**DOI:** 10.1002/ece3.3399

**Published:** 2017-09-18

**Authors:** Jianping Wu, Weixin Zhang, Yuanhu Shao, Shenglei Fu

**Affiliations:** ^1^ Institute of Ecology and Environmental Sciences Nanchang Institute of Technology Nanchang China; ^2^ Key Laboratory of Geospatial Technology for the Middle and Lower Yellow River Regions College of Environment and Planning Henan University Kaifeng China; ^3^ Key Laboratory of Vegetation Restoration and Management of Degraded Ecosystems South China Botanical Garden Chinese Academy of Sciences Guangzhou China

**Keywords:** earthworm–plant interaction, phospholipid fatty acids, *Pontoscolex corethrurus*, soil microbial community, soil respiration

## Abstract

Earthworms and plants greatly affect belowground properties; however, their combined effects are more attractive based on the ecosystem scale in the field condition. To address this point, we manipulated earthworms (exotic endogeic species *Pontoscolex corethrurus*) and plants (living plants [native tree species *Evodia lepta*] and artificial plants) to investigate their combined effects on soil microorganisms, soil nutrients, and soil respiration in a subtropical forest. The manipulation of artificial plants aimed to simulate the physical effects of plants (e.g., shading and interception of water) such that the biological effects of plants could be evaluated separately. We found that relative to the controls, living plants but not artificial plants significantly increased the ratio of fungal to bacterial phospholipid fatty acids (PLFAs) and fungal PLFAs. Furthermore, earthworms plus living plants significantly increased the soil respiration and decreased the soil NH_4_^+^‐N, which indicates that the earthworm effects on the associated carbon, and nitrogen processes were greatly affected by living plants. The permutational multivariate analysis of variance results also indicated that living plants but not earthworms or artificial plants significantly changed the soil microbial community. Our results suggest that the effects of plants on soil microbes and associated soil properties in this study were largely explained by their biological rather than their physical effects.

## 1 INTRODUCTION

Earthworms, which are regarded as engineers in soil ecosystems (Blouin et al., 2013; Edwards, 2004), play important roles in terrestrial biogeochemical cycles as a consequence of their feeding, burrowing, and production of casts (Coleman, Crossley, & Hendrix, 2004; Edwards, 2004). By feeding on litter on the soil surface or within the soil, earthworms usually enhance litter decomposition and promote soil nitrogen mineralization (Baker, 2007; Dechaine, Ruan, Sanchez‐de Leon, & Zou, 2005) and thus facilitate plant growth (Andriuzzi, Pulleman, Schmidt, Faber, & Brussaard, 2015; Johnson, Staley, McLeod, & Hartley, 2011). By altering the litter and soil conditions (Hättenschwiler & Gasser, 2005), earthworms also greatly affect the soil microbial community and the activity of microbial enzymes (Jana et al., 2010; Tao et al., 2009; Zhang et al., 2010). The changes in the microbial community and microbial enzyme activity likely influence soil functioning and processes (Lavelle, Lattaud, Trigo, & Barois, 1995; Lavelle & Martin, 1992). For example, one recent meta‐analysis reported that earthworms significantly increase soil emission of greenhouse gases and contribute 16% of the net global warming potential of soil (Lubbers et al., 2013).

The effects of earthworms on soil properties may be regulated by plants (Fischer et al., 2014; Sánchez‐de León & Zou, 2004). For instance, the diversity and productivity of plant community can greatly affect the earthworm community (Eisenhauer et al., 2009). Gormsen et al. (2004) also indicated that earthworm biomass was related to the diversity of plant community and to the traits of plant species. For example, legume species biomass was positively related to earthworm biomass, but nonlegume species did not show such a trend in the same condition. In contrast, earthworms can also affect the plant community structure by changing the plant seed germination and seedling growth (Laossi, Noguera, Decaens, & Barot, 2011).

Plants simultaneously influence the biological and physical characteristics of soil (Bardgett & Wardle, 2010). Through litter input, root growth, root exudation, and transpiration, plants can exert effects on the biological characteristics of soil. In addition, as a consequence of shading, water retention, and pore formation, plants change the physical characteristics of soil. Many of these studies were based on short time frames with repacked soil and in the absence of plants; therefore, more studies are needed about how plant biological and physical effects interact with earthworms, and thus, how these interactions influence ecosystem processes in general and particularly soil respiration (Fu, Zou, & Coleman, 2009; Lubbers et al., 2013).

We performed a field experiment with earthworms, living plants, and artificial plants. An endogeic earthworm, *Pontoscolex corethrurus*, which is a widespread exotic earthworm in tropical and subtropical regions in China, was the focus in this study (Zhang, Li, Guo, & Liao, 2005). Earthworm *Pontoscolex corethrurus* has a high tolerance for various environmental conditions and can take advantage of disturbances created by human activities (Lapied & Lavelle, 2003; Lavelle et al., 1987; Marichal et al., 2010). Artificial plants were included in a parallel study to help distinguish the biological effects of plants from the physical effects of plants. The canopy of artificial plants can shade the soil surface and buffer rainfall or throughfall. The purpose of this study was to investigate the effects of earthworms on the soil microbial community and the associated soil properties. Considering that the soil in our study site was resource‐limited as the plantations were established on the strongly degraded soil during 1980s (Yu & Peng, 1995), we hypothesized that (1) earthworm effects on soil carbon emission and soil available nitrogen will be influenced by living plants; (2) the biological effects of plant would be larger than the physical effects of plants.

## 2 MATERIALS AND METHODS

### 2.1 Site description

This study was conducted at the Heshan National Field Research Station of the Forest Ecosystem, which is located in Heshan County, Guangdong, China. The climate in this region is subtropical monsoon with a hot, humid summer and a cold, dry winter. From 2004 to 2009, the mean annual precipitation was 1534 mm, and the mean annual temperature was 22.5°C. The soil is an Acrisol (FAO, 2006). The *Acacia auriculiformis* plantation used in this study was established in 1984. The main understory species in this plantation were *Evodia lepta*,* Dicranopteris dichotoma*,* Rhodomyrtus tomentosa*,* Litsea cubeba*, and *Ilex asprella*. The mean diameter at breast height of the *A. auriculiformis* trees was 17.2 cm with the canopy coverage of approximately 50%.

### 2.2 Experimental design

In December 2007, we established a “Soil Animal Removal Experimental Study (SARES)” using 24 plots (1 m × 2 m) under the canopy of an *Acacia auriculiformis* plantation. An 80‐cm‐deep trench was formed around each plot to prevent intrusion of roots from the outside. PVC boards (0.5 cm thick, 2 m long, and 1 m wide) were then inserted into the vertical cuts to further isolate each plot; the boards extended to the bottom of the trench and 20 cm above the soil surface to prevent earthworms from moving between plots. Plants in all plots were removed by hand before the treatments were applied.

The experiment had a randomized block design with four replicates of six treatments. The six treatments included the following: living plants (LP), earthworms plus living plants (E+LP), artificial plants (AP), earthworms plus artificial plants (E+AP), and neither living plants nor artificial plants (C, the control where earthworms were removed by electrical shocking). When earthworms were added into control treatment (C), then the treatment was named C+E. For treatment LP, we planted seven seedlings of typical native species (*Evodia lepta*) per plot in 2007. In May 2009, the average diameter at the seedling base was 1.6 cm, the average height was 1.0 m, and the average seedling canopy was 0.7 m × 0.9 m. The seedlings occupied approximately 75% of the plot area. For treatment AP, artificial plants were constructed in 2009 by attaching plastic branches and plastic leaves to the stems of dead *E. lepta* plants (collected outside plots without roots, inserted plants were about 15 cm), which were used to simulate the aboveground mass and structure of living *E. lepta* plants. For treatment E, we collected the endogeic earthworm *Pontoscolex corethrurus* at nearby sites in May 2009. These earthworms were washed in running water and then added to the litter layer on the soil surface at a rate of 100 individuals per m^2^, which is in the range reported in other studies performed in the tropical regions (Coq, Barthès, Oliver, Rabary, & Blanchart, 2007; Lapied & Lavelle, 2003; Marichal et al., 2012).

**Figure 1:**
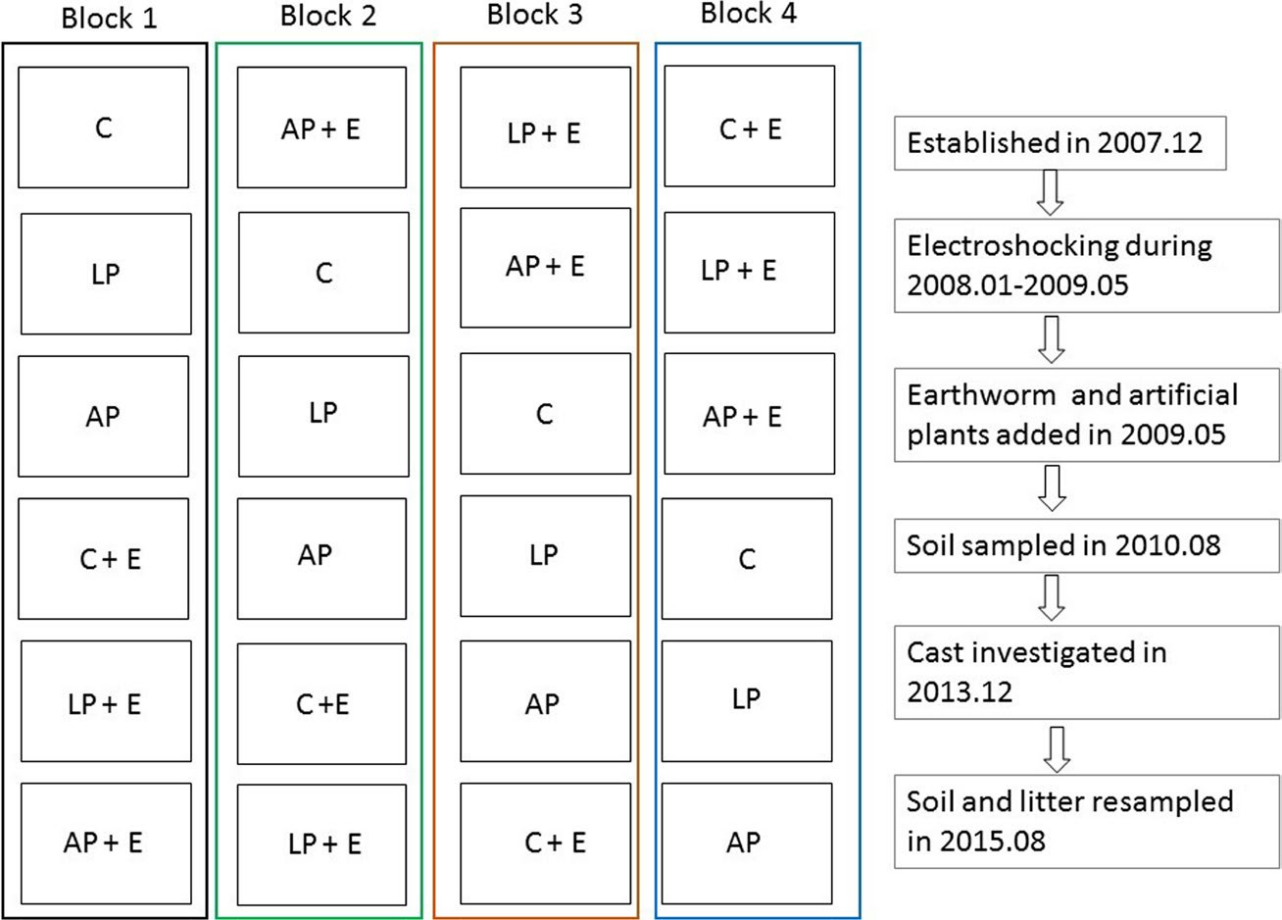
Experimental design figure that shows the manipulations performed for each treatment, timing of manipulation, and timing of measurement

The manipulations performed for each treatment, timing of manipulation, and timing of measurement were shown with experimental design figure (Figure 1). Before the earthworms were added in May 2009, electroshocking, a nondestructive method was used to reduce earthworms in all plots (Bohlen & Edwards, 1995; Liu & Zou, 2002; Szlavecz et al., 2013). The electro‐shocking system comprises three parts: battery, transformer, and electrode (Fig. S1). Electro‐shocking manipulation was performed once per month from January 2008 to May 2009. All earthworms at the soil surface were removed from the plots. There are two major earthworm groups in our study site: pheretimoid (including genera of *Amynthas* and *Metaphire*, both are native species) and *P. corethrurus* (the only exotic species) (Zhang et al., 2005). As native pheretimoid earthworms are more sensitive to electroshocking compared to *P. corethrurus*, most of the living pheretimoid earthworms came out and were eliminated after electroshocking. In contrast, *P. corethrurus* respond slowly to electroshocking and thus some individuals may persist in the soil. *P. corethrurus* was added after electroshocking.

Because the “Soil Animal Removal Experimental Study (SARES)” platform is still running, it was not possible to conduct a destructive sampling for earthworm abundance determinations. However, larger volumes and masses of earthworm casts were observed in plots with added earthworms, regardless of whether plants were present. We examined the mass of earthworm casts on the soil surface in the control, living plant (LP), earthworm addition (E), and living plant plus earthworm addition (EP) treatments in December 2013. The mass of earthworm casts was used as an indicator of earthworm population size and earthworm activities (Hauser, Norgrove, Asawalam, & Schulz, 2012; Lavelle et al., 1998).

### 2.3 Sampling collection and analyses

Three soil cores (3 cm diameter, 20 cm depth) were collected from each plot in August 2010, approximately 3 years after the plots were established and 15 months after earthworms were added. The soil cores from each plot were mixed and then divided into half; one part was used for determination of soil physicochemical characteristics, and the other part was used for phospholipid fatty acid (PLFAs) analysis. For the determination of physicochemical characteristics, fresh soil was passed through a 2‐mm sieve; the remaining roots and stones were removed by hand. The soil for PLFA analysis was stored at −20°C.

Soil respiration was measured in August 2010 between 9:00 a.m. and 12:00 a.m. using an LI‐8100 automated soil CO_2_ flux system (LI‐COR Inc., Lincoln, NE, USA). To measure soil respiration, three PVC collars (20 cm diameter and 5 cm high) were placed at 2 cm depth in each plot, and living plants in the soil collars were removed by hand. The soil temperature at 5 cm depth was measured when respiration was measured using a probe that was attached to an automated CO_2_ measurement device. Dissolved organic carbon (DOC) and dissolved nitrogen in filtered 0.5 M K_2_SO_4_ extracts of fresh soil samples were measured using a TOC analyzer (TOC‐VCPH Shimadzu Corp., Japan*)*. NH_4_^+^‐N and NO_3_^−^‐N in filtered 2 M KCL extracts of fresh soil sample were measured using a flow injection autoanalyzer (FIA, Lachat Instruments, USA). Soil moisture (g of water per 100 g dry soil) was measured gravimetrically by drying fresh soil at 105°C to constant weight. The N contents were examined after micro‐Kjeldahl digestion using a flow injection autoanalyzer (Liu, 1996).

The soil microbial community was characterized using PLFAs analysis as described by Bossio & Scow, (1998). The concentration of individual fatty acids was determined as nmol per g of dry soil, and standard nomenclature was used (Tunlid, Hoitink, Low, & White, 1989). Bacteria were considered to be represented by 10 PLFAs (i15:0, a15:0, 15:0, i16:0, 16: 1ω7, i17:0, a17:0, 17:0, cy17:0, and cy19:0), and fungi were considered to be represented by the 18:2ω6 PLFA (Bossio & Scow, 1998; Frostegård & Bååth, 1996). Other PLFAs such as 16:1ω9c, 16:0, 17:1ω8c, 18:1ω9c, and 18:3ω3c were also used to analyze the composition of the microbial community. The ratio of 18:2ω6 to total bacterial PLFAs was used to estimate the ratio of fungal to bacterial biomass (F: B) (Bardgett, Hobbs, & Frostegård, 1996; Frostegård & Bååth, 1996). All of the PLFAs indicated above were considered to be representative of the total PLFAs and analyze the soil microbial community (Zhang et al., 2013).

In August 2015, we resampled soils and collected the litter of *A. auriculiformis* in the treatments of plants (LP), earthworms plus living plants (E+LP), artificial plants (AP), and earthworms plus artificial plants (E+AP). Meanwhile, the fresh leaf and litter of *E. lepta* were also collected in the treatments of living plants (LP) and earthworms plus living plants (E+LP). Both fresh leaf and litter were oven‐dried at 70°C and ground for measurement. N contents were measured for the second samples.

### 2.4 Data analyses

**Figure 2:**
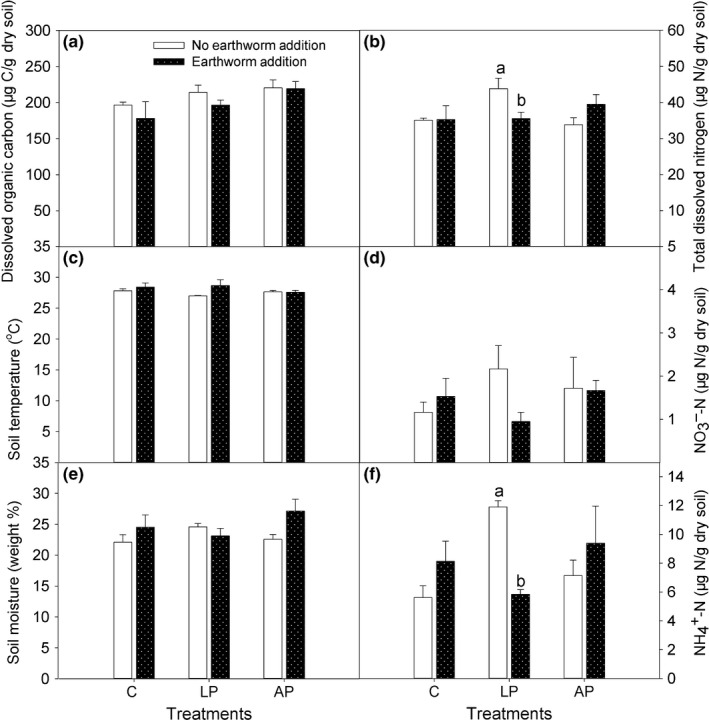
Soil characteristics (dissolved organic carbon, total dissolved nitrogen, soil temperature at 5 cm depth, NO_3_^−^‐N, soil moisture, and NH_4_^+^‐N) were affected by the addition of earthworms to control plots (C), plots with living plants (LP), and plots with artificial plants (AP). Values are expressed as the means ±1 *SE*,* n *=* *4. For comparison of the plots with and without earthworms within the C, LP, and AP treatments, bars with different letters are significantly different at *p *<* *.05. The sampling time was 2010

**Figure 3:**
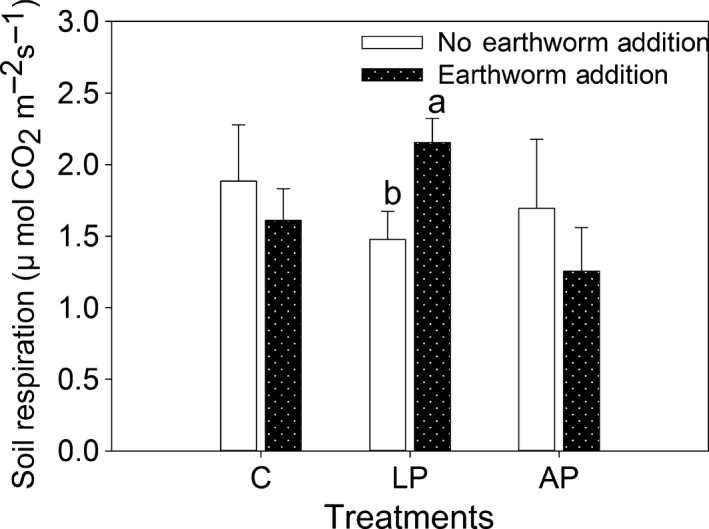
Soil respiration as affected by the addition of earthworms to control plots (C), plots with living plants (LP), and plots with artificial plants (AP). Values are means ±1 *SE*,* n *=* *4. For comparison of plots with and without earthworms within C, LP, and AP treatments, bars with different letters are significantly different at *p *<* *.05. The sampling time was 2010

**Figure 4:**
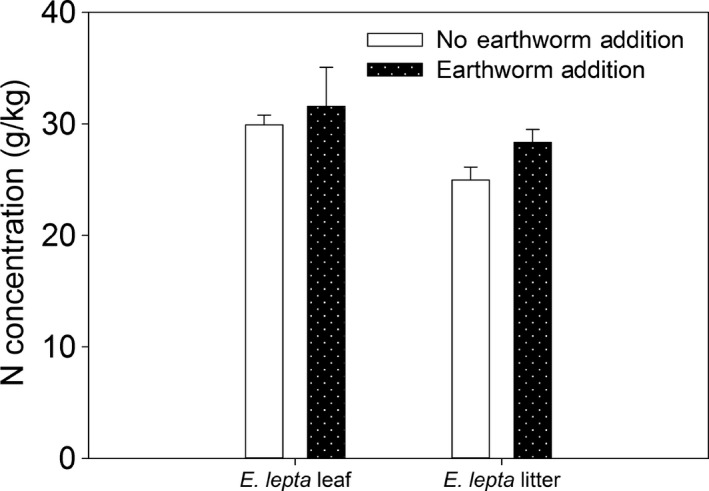
N contents of fresh and litter of *Evodia lepta* were affected by the addition of earthworms. Values are expressed as the means ±1 *SE*,* n *=* *4. For comparison of the plots with and without earthworms, bars without letter are not significantly different at *p *<* *.05. The sampling time was 2015

**Figure 5:**
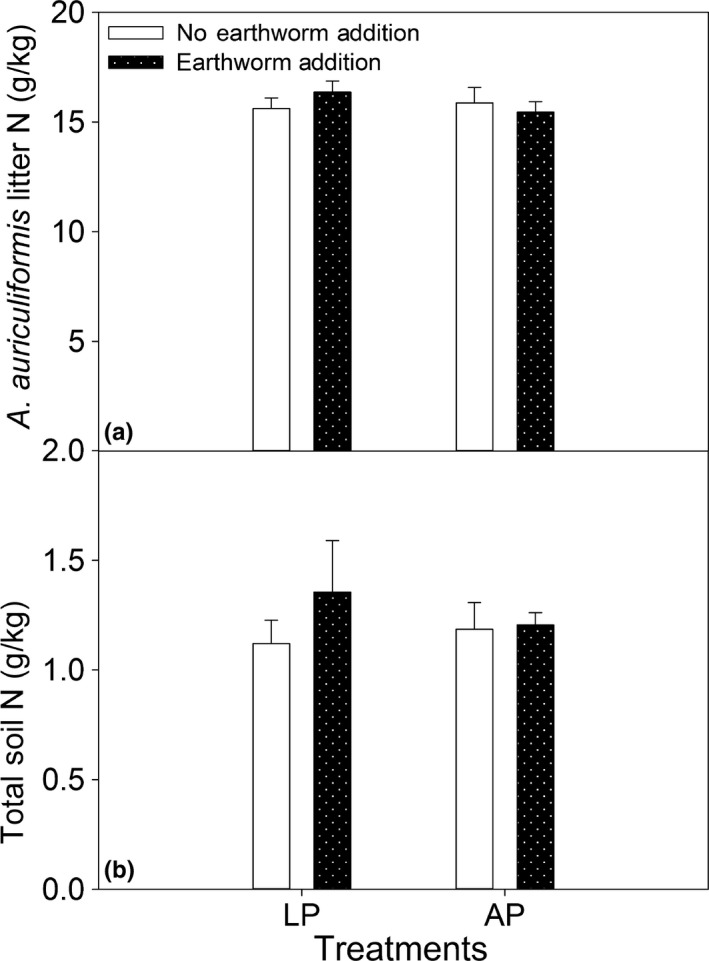
N contents of soil and litter of *Acacia auriculiformis* were affected by the addition of earthworms to plots with living plants (LP) and plots with artificial plants (AP). Values are expressed as the means ± 1 *SE*,* n *=* *4. For comparison of the plots with and without earthworms within the LP and AP treatments, bars without letter are not significantly different at *p *<* *.05. The sampling time was 2015

**Figure 6:**
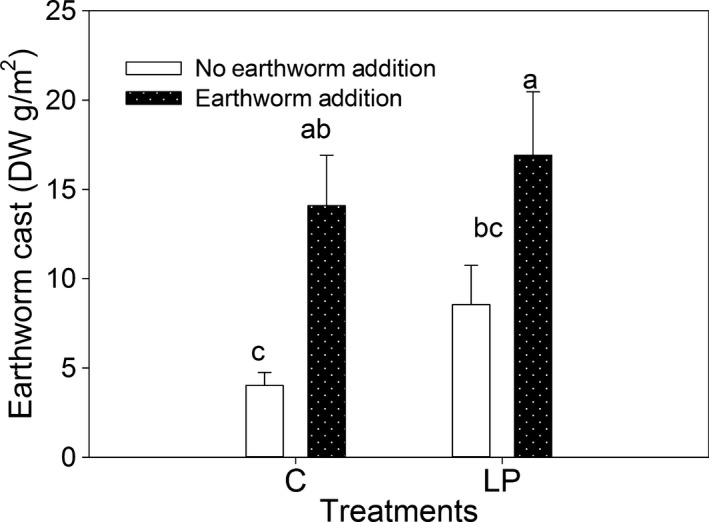
Dry weight (DW g/m^2^) of the earthworm casts in the treatments of controls (C), living plants (LP), earthworm addition (C+E), and living plants plus earthworm addition (E + LP) in December 2013. The mass of earthworm casts was used to indicate earthworm abundance. To determine the weight of the earthworm casts, we selected one 30 cm × 30 cm subplot in each replicated plot, and the earthworm casts were collected, freeze‐dried, and weighed. Values are expressed as the means ± 1 *SE*,* n *=* *4, and bars with different letters are significantly different at *p *<* *.05. The sampling time was 2013

**Figure 7:**
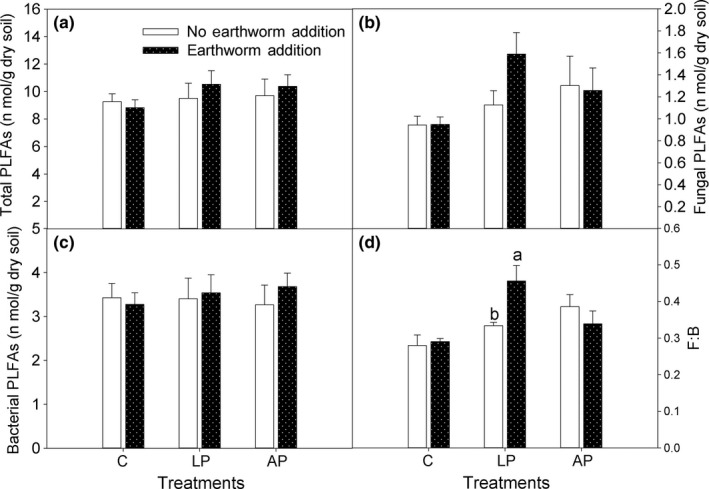
Soil microbial PLFAs as affected by the addition of earthworms to control plots (C), plots with living plants (LP), and plots with artificial plants (AP). Values are expressed as the means ±1 *SE*,* n *=* *4. For comparison of plots with and without earthworms within the C, LP, and AP treatments, bars with different letters are significantly different at *p *<* *.05. The sampling time was 2010

We used two‐way anova to test for the effects of earthworms, plants, and their interactions on the soil microbial characteristics and environmental factors. Here, plant effects divided into living plant and artificial plant effects. The comparisons of living plants versus controls, living plants versus artificial plants, and artificial plants versus controls were also calculated using Tukey's honest significant difference test (Table 1). The paired sample *t* test was used to determine the difference for three treatment groups (C vs. C+E, LP vs. LP+E, and AP vs. AP+E) (Figures 2, 3, 4, 5 and 7). One‐way anova was used to analyze the differences in earthworm cast mass among the control, living plant, earthworm addition, and living plant plus earthworm treatments (Figure 6). The block was considered a random factor in this study. These statistical analyses were performed using SPSS 15 (SPSS, Inc., Chicago, IL, USA). Significance was determined at the 0.05 level.

**Table 1:** Effects of plants (P), earthworms (E), and their interactions on soil microbial PLFAs and soil properties as indicated by *p* values from two‐way anovas. The *p* values of multiple comparisons between living plants versus controls (LP vs. C), living plants versus artificial plants (LP vs. AP), and artificial plants versus controls (AP vs. C) are also shown in the table. See Figure 3 for abbreviations. The bold fonts in table mean the significant effect. The sampling time was 2010

Variables	P	E	P × E	LP versus C	LP versus AP	AP versus C
Total PLFAs	0.32	0.49	0.63	0.48	0.96	0.33
Bacterial PLFAs	0.62	0.48	0.71	0.81	0.94	0.60
Fungal PLFAs	**0.04**	0.22	0.43	**0.03**	0.54	0.22
F:B	**0.02**	0.26	0.44	**0.01**	0.20	0.38
DOC	0.054	0.23	0.74	0.34	0.48	**0.04**
TDN	0.21	0.69	**0.04**	0.19	0.46	0.82
SR	0.53	0.96	0.19	0.97	0.54	0.67
NO_3_^−^‐N	0.73	0.42	0.21	0.88	0.95	0.71
NH_4_^+^‐N	0.34	0.70	**0.01**	0.33	0.89	0.56
ST	0.63	0.10	0.26	0.86	0.90	0.61
SMC	0.54	0.12	0.12	0.92	0.75	0.52

We performed permutational multivariate analysis of variance (permanova) to determine the effects of plants, earthworms, and their interactions on the composition of the soil microbial community. permanova is nonparametric statistical method for ecological multivariate data sets (Anderson, 2001), which is an appropriate way to analyze the community data because it allows for the testing of main effects (plant and earthworm) and their interactions on soil microbial community. permanova was performed on Euclidean distances using PC‐ORD 5.0 (McCune & Mefford, 2006). As the plant factor included living plant and artificial plant, so we divided the P effects into LP effect and AP effect in the tables when conducting multiple comparisons.

## 3 RESULTS

### 3.1 Physical responses

The effects of treatments usually had no effect on the soil temperature and soil moisture in 2010 (Figure 2c,e). Two‐way anovas showed that the effects of plants, earthworms, and the interaction between earthworms and plants were not significant for the soil temperature and soil moisture (Table 1).

### 3.2 Chemical responses

In treatments without earthworm addition, the presence of living plants (LP) resulted in the highest values of total dissolved nitrogen, NO_3_^−^‐N, and NH_4_^+^‐N in 2010 (Figure 2b,d,f). When compared with LP treatment, the addition of earthworms to plots with living plants (E + LP), however, reduced total dissolved nitrogen by 19% (*p *=* *.05; Figure 2b), reduced NO_3_^−^‐N by 56% (*p *=* *.07; Figure 2d), reduced NH_4_^+^‐N by 51% (*p *<* *.001; Figure 2f), and increased soil respiration (*p *=* *.04; Figure 3). In addition, the effects of plants, earthworms, and the interaction between earthworms and plants were not significant for soil characteristics except that the E × P interaction significantly affected TDN and NH_4_^+^‐N concentrations (Table 1).

The samples collected in 2015 showed that the addition of earthworms did not significantly enhance N contents of fresh leaf and litter of *E. lepta* (Figure 4). The addition of earthworms did not affect the N contents of soil and litter of *A. auriculiformis* (Figure 5).

### 3.3 Biological responses

The results showed that level of earthworm casts was highest in the living plant plus earthworm condition, and followed by earthworm addition, living plants, and control in 2013 (Figure 6), suggesting that added earthworms lived in the field plots.

The addition of earthworms to plots with living plants (E + LP), however, increased the ratio of fungal PLFAs to bacterial PLFAs by 37% (*p *=* *.03; Figure 7d) and tended to increase fungal PLFAs (*p *=* *.096; Figure 7b). According to two‐way anovas, the effects of plants were significant for the fungal PLFAs (*p *=* *.04) and the ratio of fungal PLFAs to bacterial PLFAs (*p *=* *.02), which were significantly higher in LP plots (*p *=* *.03 and *p *=* *.01, respectively) than in C plots (Table 1). The earthworm effect and the interaction between earthworms and plants were not significant for the microbial PLFAs (Table 1).

permanova showed that the soil microbial community was strongly affected by the plant treatment (*p *=* *.003, Table 2) but not by the earthworm treatment (*p *=* *.07, Table 2). There was no interaction between earthworms and plants on the soil microbial community (Table 2). The results of pairwise comparisons showed that living plants developed a different soil microbial community compared to the artificial plant and control treatments. There was no significant difference between the control treatment and artificial plant treatment.

**Table 2:** Effects of plants (P), earthworms (E), and their interactions on the soil microbial community composition as performed by permanova. Pairwise comparisons between living plants versus controls (LP vs. C), living plants versus artificial plants (LP vs. AP), and artificial plants versus controls (AP vs. C) are also shown in the table. The bold fonts in table mean the significant effect. The sampling time was 2010

Source	*df*	SS	MS	*F*	*p*
P	2	3057.30	1528.60	4.91	**.003**
E	1	1053.80	1053.80	3.39	.07
P × E	2	743.30	371.65	1.19	.38
Residual	18	5601.90	311.21		
Total	23	10456			

Comparisons	*t*	*p*
LP versus C	2.42	**.011**
LP versus AP	2.64	**.006**
C versus AP	1.53	.17

## 4 DISCUSSION

Our results emphasize that the pan‐tropical widespread earthworm species of *P. corethrurus* only show significant effects on soil carbon and nitrogen dynamics and soil microbial community in plots with living plants in the studied subtropical plantation. These findings indicate that plants would be the primarily regulator of earthworm‐related ecological processes (Johnson et al., 2011; Velásquez et al., 2012).

Firstly, earthworms affected soil respiration when living plants were present. However, the combination of earthworms and artificial plants did not significantly influence soil respiration. On one hand, soil respiration was not greatly affected by the addition of artificial plants. On the other hand, the artificial plants did not affect soil moisture or soil temperature. The failure of the artificial and living plant treatments to affect soil moisture and especially soil temperature likely occurred because most of the solar radiation was intercepted by the canopies of *A*. *auriculiformis* trees, *E. lepta* seedlings, and artificial plant leaves. Therefore, there was little effect of the solar radiation hitting the soil surface. Our mesocosm plots were under the *A. auriculiformis* canopy, but the input of Acacia litter did not contribute to the results because the quantity and quality of litter that were input into the plots were similar in this study. The effects of artificial plants, however, would be underestimated as we only considered the aboveground aspects but not belowground aspects, of which the root systems would also affect physical properties. Furthermore, the electroshocking would not remove all earthworms especially in the deep soil layers, so the effects of earthworms were likely underestimated.

The addition of earthworms to the control plots did not significantly reduce soil respiration, although the values showed a slightly decrease. These results were partially consistent with the results obtained by Six, Bossuyt, Degryze, and Denef (2004), which reported that earthworms had negative effects on soil respiration by physically protecting soil carbon. Our recent research also indicated that earthworms facilitate carbon sequestration by accelerating carbon activation leading to larger carbon stabilization compared with carbon mineralization (Zhang et al., 2013). In contrast, the amount of soil respiration increased by 45.9% when earthworms were added along with living plant treatments. An increase in soil respiration when plants grow with earthworms was also evident in a short‐term experiment performed at our study site (Gao et al., 2010). Soil respiration would be affected by plant–soil interactions, indicating that further research is needed.

Secondly, earthworm effects on soil nitrogen dynamics were regulated by plants. Our results showed that the endogeic earthworm *P. corethrurus* significantly decreased the level of available nitrogen in soil when living plants were present. We consider that this decrease might be that the increased mineralized nitrogen by earthworms was less than the earthworm‐induced increase of nitrogen uptake by plants. In a recent study, for example, the endogeic species *Aporrectodea caliginosa* increased soil NO_3_^−^‐N levels by 31% and soil NH_4_^+^‐N levels by 4% (McDaniel, Stromberger, Barbarick, & Cranshaw, 2013). While, plants would take advantage of nitrogen that mineralized by earthworms and the associated microorganisms and thus decrease the soil nutrient concentration (González & Zou, 1999; Sánchez‐de León & Zou, 2004)**.** Although the N concentrations of soil and *E. letpa* leaf did not change significantly after earthworm addition, the addition of *P. corethrurus* earthworms may still increase plant production by stimulating nitrogen mineralization (Fonte, Quintero, Velásquez, & Lavelle, 2012; González & Zou, 1999; Lafont et al., 2007; Pashanasi, Lavelle, Alegre, & Charpentier, 1996).

It was worth noting that the contents of total dissolved nitrogen in the living plant treatment were higher than that in the control treatment when earthworms were absent. There were two potential reasons. Given that soil N leaching was common in the study region (Fang et al., 2009), soil nitrogen could be more readily leached by rainfall and throughfall in the control treatment. The other was that the litter input of *E. letpa* and *A. auriculiformis* in the living plant treatment would improve the microenvironment condition for soil microbial community and thus enhance nitrogen availability, in which the largest difference of soil available N can reach to 56.37 mg/kg under different canopies (Xing, Huang, An, & Zhang, 2013).

Thirdly, the results of permutational multivariate anova showed that the living plant was a dominant factor and earthworms played a secondary role in affecting soil microbial community composition, which was also supported by Blanchart, Albrecht, Chevallier, and Hartmann (2004). Although the quantity of total microbial PLFAs was unaffected by the treatments in this study, the combination of earthworms and living plants significantly increased the ratio of fungal to bacterial PLFAs. In previous studies, fungal growth or fungal biomass was increased by the planting of *E. lepta* seedlings (Gao et al., 2010) or by earthworm addition (Yu, Cheng, & Wong, 2005). We therefore consider that earthworm addition enhanced the fungal activities and contributed to the increase in soil respiration only in the plots containing living plants. Recent reports showed that soil fungi can also enhance the decomposition of soil organic matter (Cheng et al., 2012) and that the presence of *P. corethrurus* promoted soil respiration and litter decomposition in tropical soils (Chapuis‐Lardy, 2010; Zhang et al., 2010), which supported our assumption.

## 5 CONCLUSION

Based on the field experiment in the subtropical forest, the analysis of soil properties shows that the combined effect of earthworms and plants decreased the available soil nitrogen and increased the soil CO_2_ emissions when compared with only plant treatment. In addition, our findings indicate that plants but not earthworms significantly changed the soil microbial community composition. These results suggest that plants facilitate the effects of exotic earthworm *Pontoscolex corethrurus* on soil carbon and nitrogen dynamics and soil microbial community in the subtropical field ecosystem. The plant effects on soil microbes in this study are largely explained by their biological rather than their physical effects although we recognize that the treatment of artificial plant has a limitation as both aboveground part and roots would affect physical properties.

## CONFLICT OF INTEREST

The authors declare no competing financial interests.

## AUTHOR CONTRIBUTIONS

JPW, SLF, WXZ, and YHS conceived, designed, and performed the experiments. All authors wrote the manuscript and analyzed the data.
