# Supplementary material for: Plant‐facilitated effects of exotic earthworm Pontoscolex corethrurus on the soil carbon and nitrogen dynamics and soil microbial community in a subtropical field ecosystem
Source: Ecol Evol. 2017 Sep 18;7(21):8709–18. doi: 10.1002/ece3.3399 (PMC5677479; doi:10.1002/ece3.3399)

**S-Figure 1.** Earthworm manipulation in the field by electric shock. The electro-shocking system comprises three parts: battery, transformer and electrode as shown.


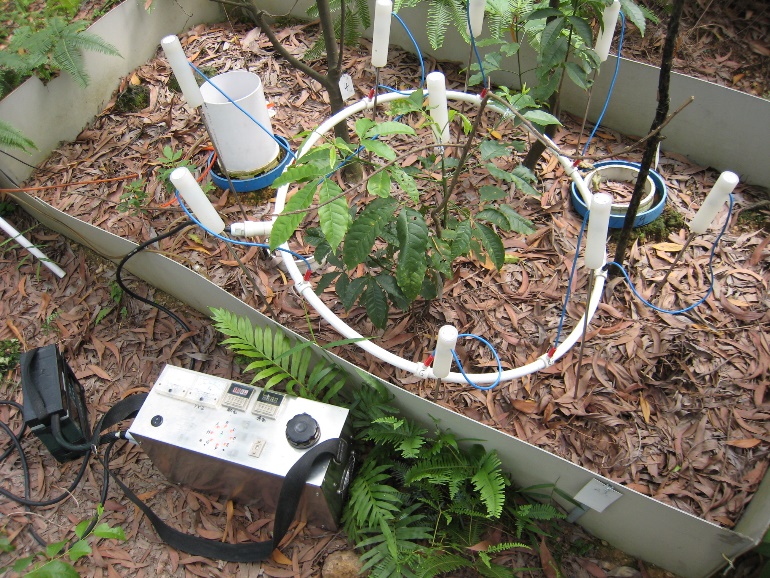

Supplement: Supplementary file 1 [file ECE3-7-8709-s001.docx]
